# Elevated plasma level of visfatin/pre-b cell colony-enhancing factor in male oral squamous cell carcinoma patients

**DOI:** 10.4317/medoral.18574

**Published:** 2012-12-10

**Authors:** Tsai Yu-Duan, Wang Chao-Ping, Chen Chih-Yu, Lin Li-Wen, Lin Tsun-Mei, Hsu Chia-Chang, Chung Fu-Mei, Lin Hsien-Chang, Hsu Hsia-Fen, Lee Yau-Jiunn, Houng Jer-Yiing

**Affiliations:** 1 Division of Neurology, Department of Surgery and; 2 Division of Cardiology and; 3 Division of Gastroenterology and Hepatology, Department of Internal Medicine and; 4 Department of Dentistry and; 5 Department of Medical Laboratory, and; 6 Department of Medical Research, E-Da Hospital, I-Shou University, Kaohsiung, 82445 Taiwan; 7 Li-Tzung biotechnology, INC. Kaohsiung, 80143 Taiwan; 8 Department of Medical Nutrition, Institute of Biotechnology and Chemical Engineering I-Shou University, Kaohsiung, 82445 Taiwan; 9 Lee’s Endocrinologic Clinic Pingtung, 90000 Taiwan

## Abstract

Objectives: Visfatin, also known as nicotiamide phosphoribosyltransferase or pre-B cell colony enhancing factor, is a pro-inflammatory cytokine whose serum level is increased in various cancers. In this study, we investigated whether plasma visfatin levels were altered in patients with oral squamous cell carcinoma (OSCC). The relationship between plasma visfatin levels and the pretreatment hematologic profile was also explored. Study Design: Plasma visfatin concentrations were measured through ELISA in OSCC patients and control subjects. A total of 51 patients with OSCC and 57 age- and body mass index (BMI)-matched control subjects were studied. All study subjects were male. Results: Plasma visfatin was found to be elevated in patients with OSCC (7.0 ± 4.5 vs. 4.8 ± 1.9 ng/ml, p = 0.002). Multiple logistic regression analysis revealed visfatin as an independent association factor for OSCC, even after full adjustment of known biomarkers. Visfatin level was significantly correlated with white blood cell (WBC) count, neutrophil count, and hematocrit (all p < 0.05). In addition, WBC count, neutrophil count, and visfatin gradually increased with stage progression, and hematocrit gradually decreased with stage progression (all p < 0.05). Conclusion: Increased plasma visfatin levels were associated with OSCC, independent of risk factors, and were correlated with inflammatory biomarkers. These data suggest that visfatin may act through inflammatory reactions to play an important role in the pathogenesis of OSCC.

** Key words:**Visfatin; oral squamous cell carcinomas; white blood cell count; neutrophil count.

## Introduction

Oral cancer is the most common malignancy observed in the head and neck area. In more than 90% of cases, oral cancer is characterized by oral squamous cell carcinoma (OSCC) (1). The high prevalence of OSCC in Asia may be due to the existence of certain lifestyle factors such as the chewing of betel quid together with or without tobacco, alcohol consumption, cigarette smoking, infection with human papilloma virus and inadequate oral hygiene (2-4). In addition, inflammatory cytokines may play an important role (5). Chronic inflammation has been causally associated with various types of cancer (6,7). Numerous studies have reported that the inflammatory infiltrate can be a strong risk factor for cancer development in chronic, inflammatory conditions. Adipose tissue produces several proteins (adipocytokines), such as TNF-alpha, IL-6, type-1 plasminogen activator inhibitor (PAI-1), adiponectin, leptin, resistin, visfatin and apelin are associated with the risk of cancer at various sites (e.g., breast, prostate gland, endometrium and colorectum) (8-11). The altered secretion of metabolically active, proinflammatory adipocytokines from adipose tissue is believed to play a key role in the mechanisms related cancer (12).

Visfatin has been proposed as being expressed in normal, inflamed, and tumor tissues (13,14). It possesses nicotinamide adenine dinucleotide (NAD) biosynthetic activity and regulates growth, apoptosis, and angiogenesis in mammalian cells (15,16). Visfatin was originally identified as a pre-B-cell colony-enhancing factor and was thought to play roles in immune response and inflammation (17-19). Thus, there is some evidence to suggest that visfatin activates proinflammatory cytokines in human monocytes (20). Additionally, serum visfatin concentration is increased in patients with sepsis, chronic kidney disease and cancer (8,21,22), which indicates that visfatin plays a pro-inflammatory role in peripheral tissues. However, until now, data regarding the role of visfatin and the association between visfatin and hematologic profile in patients with OSCC is relatively limited. To investigate the role of visfatin in OSCC, we measured pretreatment plasma visfatin level as well as pretreatment hematologic profile in a Chinese population with OSCC.

## Subject and Methods

-Patients 

A total of 51 consecutive male subjects with histologically proven OSCC were enrolled in this prospective study ( Table 1). All of the subjects were hospitalized for examination and treatment. The patients ranged in age from 33 to 89 years old (mean 53 years). The primary sites of cancer were buccal mucosa (26 cases), tongue (8 cases), retromolar (5 cases), lip (2 cases), palate (4 cases), and floor of the mouth (6 cases).

**Table 1:**
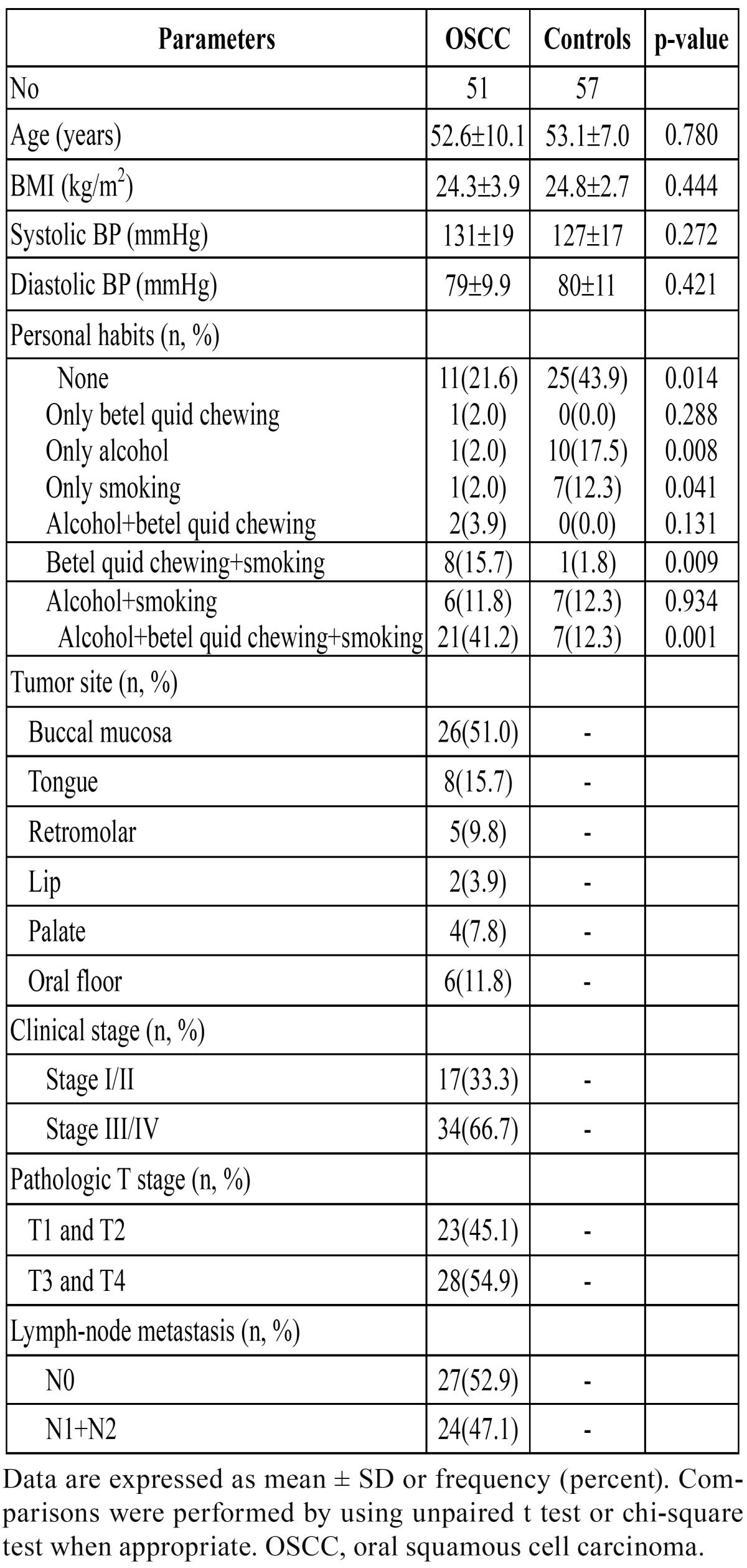
Demographic characteristics of the participants.

A TNM classification was made in accordance with the criteria for oral cancer tumors of the American Joint Committee on Cancer (AJCC) staging system (sixth edition) (23). Regarding the T-stage, 6 cases (11.8%) were classified as T1, 17 (33.3%) as T2, 9 (17.7%) as T3, and 19 (37.3%) as T4. Regarding N-stage, 27 cases (52.9%) had no cervical lymph node metastasis (N0), and 10 (19.6%) and 14 (27.5%) cases were classified as N1 and N2, respectively. Regarding M-stage, 50 cases (98.0%) not spread to distant parts of the body (M0), and 1 case (2%) was classified as M1.

Histopathologic evaluation was performed using a hematoxylin and eosin-stained preparations from the pretreatment biopsy specimens by 2 of the authors (H-C L and Y-T L), who were unaware of which patients the specimens came from. The degree of histologic differentiation was determined in accordance with the WHO criteria published in 1997: 5 cases (9.8%) had grade 1 cancer, 12 cases (23.5%) had grade 2 cancer, 5 cases (9.8%) had grade 3, and 29 cases (56.9%) had grade 4 cancer. Regarding the treatment modality, 44 patients (86.3%) underwent surgery. Of the remaining 7 patients, 1 was treated with radiation therapy and 6 with chemotherapy.

Fifty-seven healthy age- and BMI-matched volunteers were recruited as control subjects. The control subjects ranged in age from 44 to 76 years, with a mean age of 53 years ( Table 1). All of the patients and control subjects were Taiwanese (single race). All study subjects were male. None of the patients or control subjects had a history of autoimmune diseases or hepatitis. This study was approved by the Human Research Ethics Committee of our hospital, and written informed consent was obtained from each participant before enrollment.

Each subject was interviewed in person to obtain demographic information, and information on occupation, betel quid chewing, smoking history, alcohol drinking habit, and personal and family history of various cancers. Detailed information was obtained regarding each subject’s betel quid chewing, cigarette smoking, and alcohol drinking habits; we specifically queried at what age the habits began, what the average daily consumption quantity was, and at what age the habits stopped. Plasma biochemical parameters were also measured after overnight fasting including triglycerides, total cholesterol, creatinine, and glucose, which were measured by standard commercial methods on a parallel, multichannel analyzer (Hitachi7170A, Tokyo, Japan) as our previous reports (24). Peripheral complete blood cell count was determined by an automated cell counter (XE-2100 Hematology Alpha Transportation System, Sysmex Corporation, Kobe, Japan). To minimize the confounding effect of infection, subjects with a WBC count below 4.0 Χ 109/l or greater than 10.0Χ 109/l were re-checked and studied extensively to rule out the existence of chronic infections.

-Plasma visfatin measurement

All of the blood samples were drawn after overnight fasting and plasma samples were kept at -80°C for subsequent assay. The concentrations of plasma visfatin were determined by commercial enzyme immunoassay kits (Phoenix Pharmaceuticals, Belmont, CA). The intraassay coefficients of variation were 2.4-2.7% for visfatin. Samples were measured in duplicate in a single experiment.

-Statistical analyses

The data are shown as the mean ± SD. All of the statistical analyses were performed using the SAS software (release 8.0; SAS Institute, Cary, NC). The unpaired Student’s t-test was used for between-group comparisons for continuous variables, and the χ2 test was used for categorical variables. One-way analysis of variance was performed to examine the prevalence of each variable between tumor stage groups. Because the distributions of plasma visfatin, triglyceride, SGOT, SGPT, and hematocrit values were skewed, logarithmically transformed values were used for statistical analysis.

The association of visfatin with diabetes was examined by multivariate logistic regression analysis that contained: 1) visfatin, age, and BMI; 2) visfatin, age, BMI, systolic blood pressure (SBP), and diastolic blood pressure (DBP); 3) visfatin, age, BMI, SBP, DBP, total cholesterol, and triglyceride; 4) visfatin, age, BMI, SBP, DBP, total cholesterol, triglyceride, betel quid chewing, drinking, smoking status. Multivariate adjusted ORs are presented with 95% confidence interval (CI). Pearson’s correlation analyses were used to examine the correlation between plasma concentrations of visfatin and the values of other biomarkers. All of the statistical analyses were two sided, and P < 0.05 was considered significant.

Results

The demographic characteristics of our subjects are shown in  Table 1. Patients with OSCC had a significantly higher betel quid chewing accompanied with smoking and drinking and betel quid chewing accompanied with smoking rates, and lower non-personal habits (betel quid chewing, smoking, and drinking), and only drinking, and only smoking rates than control subjects. The biochemical characteristics of the study subjects are shown in  Table 2. Plasma visfatin levels were found to be elevated in patients with OSCC (7.0 ± 4.5 vs. 4.8 ± 1.9 ng/ml, P = 0.002). Patients with OSCC had significantly higher fasting glucose, SGOT, WBC, neutrophil count, monocyte count, lymphocyte count, and platelet count, and lower albumin, hemoglobin, and hematocrit levels than those of control subjects. The mean BMI, blood pressure, total-cholesterol, triglyceride, SGPT, creatinine, total protein, and red blood cell were similar in both groups.

**Table 2:**
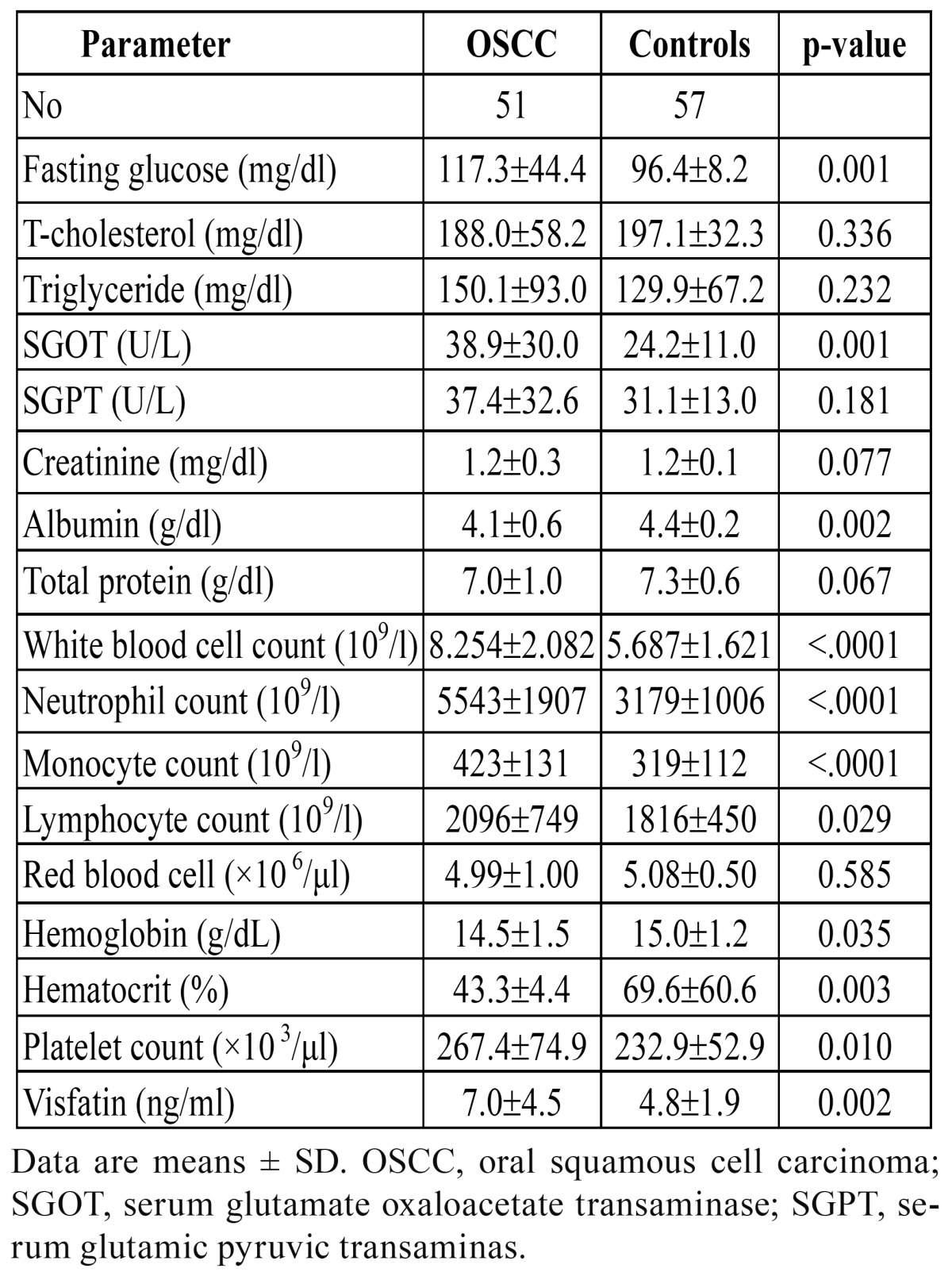
Biochemical characteristics of the study patients.

**Figure 1:**
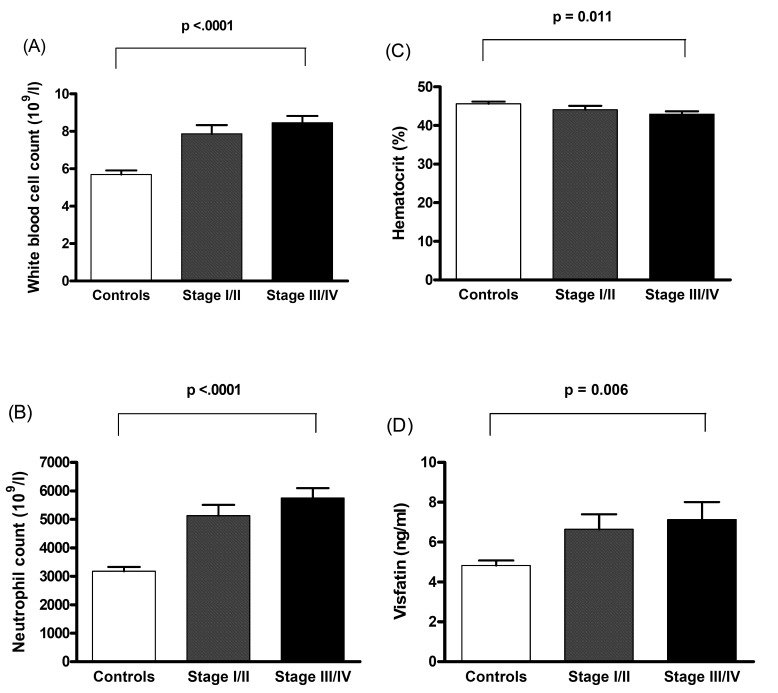
Association between white blood cell count (A), neutrophil count (B), hematocrit (C), visfatin (D), and stage progression of oral squamous cell carcinoma. Bars represent the mean ± SD. Differences between groups were analysis by one-way analysis of variance.

Plasma visfatin concentration was significantly associated with OSCC even after controlling for anthropometric variables, blood pressure, lipid profile, betel quid chewing, drinking, and smoking status ( Table 3). Using Pearson’s correlation analysis, plasma visfatin levels were correlated with WBC count, neutrophil count, and hematocrit level ( Table 4). Linear contrast analysis was conducted to evaluate the correlation between WBC count, neutrophil count, hematocrit, visfatin, and tumor stage (Fig. 1). WBC count, neutrophil count, and visfatin levels gradually increased with stage progression (P < 0.01); in contrast, hematocrit level gradually decreased with stage progression (P = 0.011).

**Table 3:**
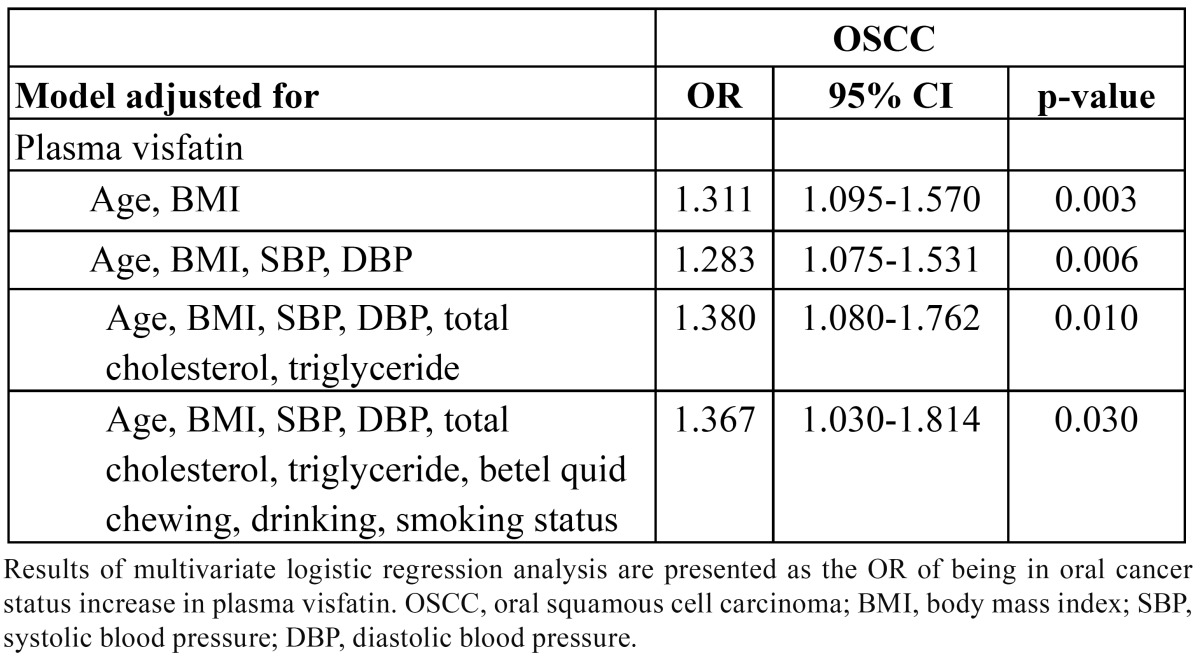
Association of plasma visfatin with oral squamous cell carcinoma in fully adjusted models.

**Table 4:**
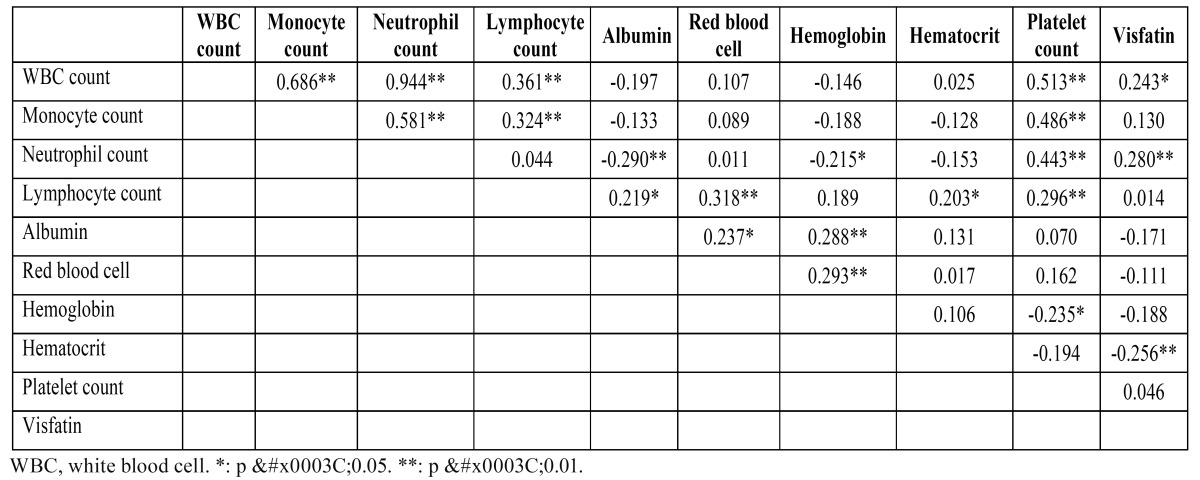
Pearson correlation coefficients of study variables in subjects studied.

## Discussion

In this study, we demonstrated that plasma visfatin concentrations were significantly elevated in a fully adjusted model in patients with OSCC. Furthermore, visfatin was gradually increased with stage progression. Moreover, we found a significant correlation between plasma visfatin and WBC count, neutrophil count, and hematocrit levels. To the best of our knowledge, this is the first report to describe elevated visfatin plasma concentrations in patients with OSCC.

The biological mechanisms involving visfatin in the pathogenesis of OSCC are not yet well understood. Visfatin is expressed not only in adipocytes and in total visceral adipose tissue (20,25,26), but also in activated immune cells such as neutrophils, macrophages, and lymphocytes (17,20,21). Visfatin activates leukocytes and induces the production of cytokines such as IL-1 β, tumor necrosis factor, and IL-6 (20). Visfatin can therefore be regarded as a proinflammatory, immunomodulating, and apopto-sis-inhibiting adipocytokine (20). Interestingly, in our study, plasma visfatin levels were found to be elevated in patients with OSCC and elevated plasma visfatin levels were associated with total WBC count and neutrophil count. Hence, on the basis of the above theories and the results of this study, we propose that visfatin should be considered as a marker of inflammation that participates in the process of OSCC.

Chronic inflammatory reactions have been shown in tumors, which may be associated with cancer progression and chemo-resistance. A previous study found that visfatin was induced by IL-1 in the human pancreatic adenocarcinoma Colo 357 cell line (27). In addition, hypoxia in the central regions of solid tumors is a leading cause of angiogenesis, a fundamental determinant of cancer progression. Bae et al. (13) demonstrated that visfatin expression was associated with expression of hypoxia-inducible factor-1a (HIF-1a) protein in breast cancer MCF7 cells and breast cancer tissue. Recently, a few studies have correlated the expression of visfatin to the clinical outcome of cancer patients (8,28,29). Reddy et al. (28) showed that levels of visfatin transcript and protein were dramatically increased in glioblastoma tissue specimens. Serum levels of visfatin in malignant astrocytomas were significantly altered compared to controls. Visfatin expression together with p53 expression was associated with shorter survival of glioblastoma patients. Nakajima et al. (8) found that visfatin was significantly associated with tumor progression in gastric cancer, suggesting that visfatin may be a useful biomarker for prediction of gastric cancer progression. In another study (29) was also showed that visfatin levels in cancer patients were significantly higher than those of controls. Stage progression significantly correlated with visfatin level. They concluded visfatin might be a useful biomarker of colorectal malignant potential and stage progression. In the present study, we found that visfatin was associated with OSCC and was gradually increased with stage progression in accordance with previous reports (8,29). In addition, our results also show visfatin concentrations being negatively correlated with hematocrit levels. A previous study demonstrates that low hematocrit levels correlate with poor survival in a group of surgically treated renal cell carcinoma patients (30). Therefore, the correlation between visfatin and hematocrit may provide an explanation for the increased risk of OSCC with high visfatin levels.

The limitations of our study include the following: (1) its cross-sectional design limits our ability to infer a causal relationship between increased plasma visfatin level and OSCC; and (2) our analyses are based on single measurements of blood visfatin, which may not reflect the relationship between visfatin levels and OSCC over time. It would be interesting to measure the serial changes in plasma visfatin concentrations in oral precancerous and oral cancer status to further clarify the role of visfatin in OSCC. In addition, although we controlled for other major cancer risk factors, the existence of unrecognized confounding variables is always possible.

In conclusion, this report shows that visfatin plasma concentrations were elevated in patients with OSCC in our Chinese study population and that there is a possible close relationship between visfatin and chronic inflammation and the development of oral cancer. Visfatin may be involved in the complex interactions involving the inflammatory or immune response. However, a large-scale prospective cohort study is necessary to resolve the potential causal relationship between visfatin and OSCC.
